# Supplementary material for: Health-related quality of life is linked to the gut microbiome in kidney transplant recipients
Source: Nat Commun. 2023 Dec 2;14:7968. doi: 10.1038/s41467-023-43431-8 (PMC10693618; doi:10.1038/s41467-023-43431-8)
Supplement: Supplementary file 1 — Supplementary Information [file 41467_2023_43431_MOESM1_ESM.pdf]

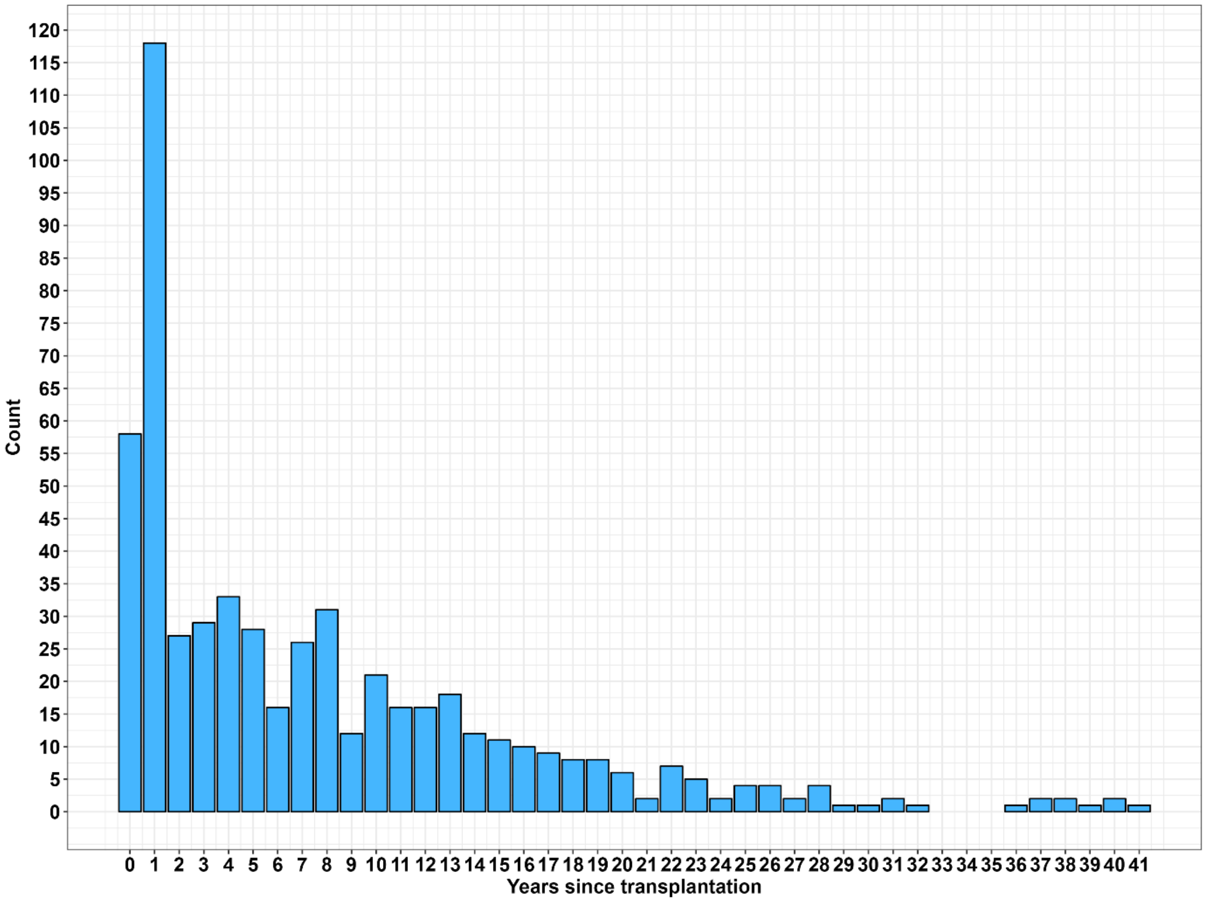

Supplementary figure 1 Years since transplantation for KTR. Bar graph depicting years since transplantation count for 507 KTR. Please note that there were 58 participants included between 10-12 months after transplantation. These 58 participants fall into the 0 years since transplantation bin in this graph.

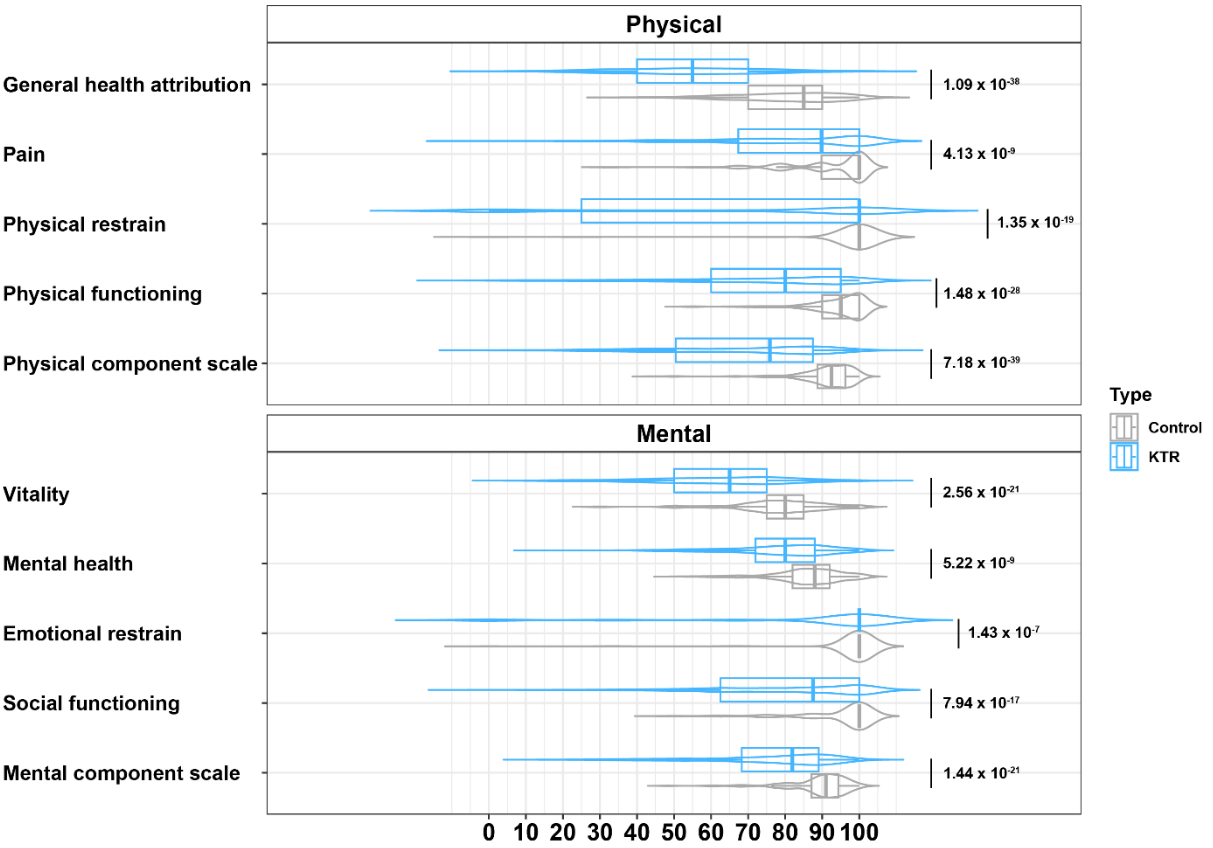

Supplementary figure 2 HRQoL for KTR and healthy controls. Each boxplot and violin plot represents HRQoL features from the SF-36-questionnaire for KTR (n=507) in blue and healthy controls from the TransplantLines cohort (n=151) in gray. All HRQoL features were significantly different between KTR and healthy controls (Wilcoxon, $P<1.43 \times 10^{-7}$)

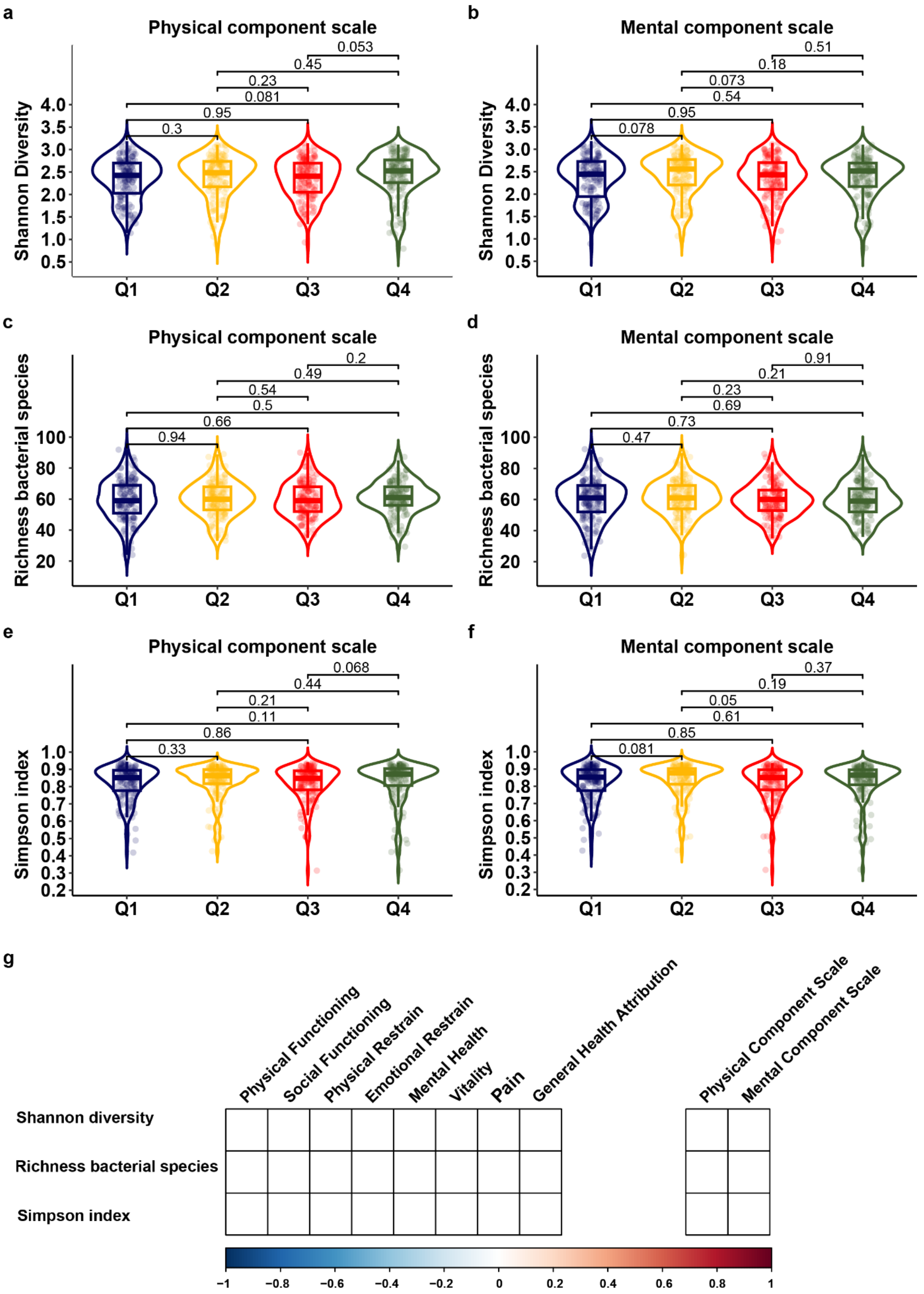

Supplementary figure 3 Diversity and HRQoL. (**a, c and e**) Violin plots depict the Shannon diversity, richness of bacterial species and the Simpson index for different quartiles of the physical component scale for 507 KTR. (**b, d and f**) Violin plots depict the Shannon diversity, richness of bacterial species and the Simpson index for different quartiles of the mental component scale. (**g**) Heatmap depicting significant (white boxes indicate a P-value>0.05) spearman correlations of the Shannon diversity index with HRQoL features from the SF-36-questionnaire.
