# Supplementary material for: Health-related quality of life is linked to the gut microbiome in kidney transplant recipients
Source: Nat Commun. 2023 Dec 2;14:7968. doi: 10.1038/s41467-023-43431-8 (PMC10693618; doi:10.1038/s41467-023-43431-8)
Supplement: Supplementary file 3 — Description of additional supplementary files [file 41467_2023_43431_MOESM3_ESM.docx]

File Name: Supplementary Data 1

Description: Characteristics of kidney transplant recipients.

File Name: Supplementary Data 2

Description: Associations potential confounders and health-related quality of life.

File Name: Supplementary Data 3

Description: Associations principal components and health-related quality of life.

File Name: Supplementary Data 4

Description: Permutational multivariate ANOVA (PERMANOVA) analysis.

File Name: Supplementary Data 5

Description: Associations between dissimilarities compared to general population controls and health-related quality of life.

File Name: Supplementary Data 6

Description: Results from Elastic-net analysis on bacterial species.

File Name: Supplementary Data 7

Description: Results from Generalized linear models bacterial species .

File Name: Supplementary Data 8

Description: Results from Elastic-net analysis on bacterial pathways.

File Name: Supplementary Data 9

Description: Results from Generalized linear models on bacterial pathways.

File Name: Supplementary Data 10

Description: Results from Elastic-net analysis on bacterial gut brain modules.

File Name: Supplementary Data 11

Description: Results from Generalized linear models on bacterial gut brain modules.
